# Modulation of cortical functional connectivity and spectrum with transcranial focused ultrasound in chronic disorders of consciousness: a case report

**DOI:** 10.3389/fpsyt.2026.1748927

**Published:** 2026-02-18

**Authors:** Shu Cheng, Xuan Xu, Lei Lei, Fanyuan Meng, Xiaojing Long, Qihui Liang, Kaixuan Luo, Xiangjun Feng, Moxian Chen, Lijuan Ao

**Affiliations:** 1 School of Rehabilitation, Kunming Medical University, Kunming, China; 2 Department of Rehabilitation Medicine, Wuhan University of Science and Technology affiliated China Resources & Wisco General Hospital, Wuhan, China; 3 Centre for Regenerative Medicine and Health, Hong Kong Institute of Science and Innovation (InnoHK), Chinese Academy of Sciences, Hong Kong, Hong Kong SAR, China; 4 Musculoskeletal Research Laboratory and Centre of Musculoskeletal Aging and Regeneration, Department of Orthopaedics and Traumatology, Faculty of Medicine, The Chinese University of Hong Kong, Hong Kong, Hong Kong SAR, China; 5 Shenzhen Institutes of Advance Technology, Chinese Academy of Sciences, Shenzhen, China; 6 Neurological Rehabilitation Department, Yunnan Rehabilitation Center for The Disabled, Kunming, Yunnan, China; 7 Department of Rehabilitation Medicine, the first affiliated hospital of Kunming Medical University, Kunming, Yunnan, China; 8 Shanghai Yangzhi Rehabilitation Hospital (Shanghai Sunshine Rehabilitation Center), Tongji University School of Medicine, Shanghai, China

**Keywords:** coma recovery scale-revised, disorders of consciousness, electroencephalogram, functional near-infrared spectroscopy, transcranial focused ultrasound

## Abstract

**Objectives:**

Chronic disorders of consciousness (DOC) represent a profound clinical challenge with few effective therapeutic options. Transcranial focused ultrasound (tFUS) is an emerging non-invasive neuromodulation technique capable of targeting deep brain structures implicated in consciousness, such as the thalamus. In this work, we present a longitudinal, multi-modal investigation into the clinical and neural outcomes following thalamic tFUS in a patient with chronic DOC.

**Methods:**

A patient in a chronic minimally conscious state underwent a course of tFUS treatment targeting the right central thalamus. The intervention was applied for 20 minutes, three times per week, over a period of four weeks (12 sessions in total). We conducted comprehensive longitudinal assessments using clinical scales (Coma Recovery Scale-Revised and Glasgow Coma Scale). Besides, resting-state functional near-infrared spectroscopy (fNIRS) to measure cortical functional connectivity, and electroencephalogram (EEG) to analyze neurophysiological dynamics.

**Results:**

The patient demonstrated a progressive and clinically significant behavioral recovery, ultimately emerging from the minimally conscious state. This recovery was accompanied by observable neural reorganization. fNIRS revealed a systematic enhancement of functional connectivity, especially within the prefrontal cortex and between prefrontal-sensorimotor networks. Concurrently, EEG analysis showed a marked, progressive suppression of pathological slow-wave (Delta and Theta) power in frontal regions. The intervention was well-tolerated with no adverse events or structural brain changes observed.

**Conclusions:**

This case provides cohesive, multi-modal evidence suggesting that targeted thalamic tFUS may be associated with a restorative process, concurrent with the normalization of dysfunctional cortical rhythms and the reintegration of large-scale brain networks that parallel the recovery of consciousness. Our findings offer preliminary insights supporting the continued investigation of tFUS as a potential therapeutic strategy for patients with severe brain injury.

## Introduction

Chronic Disorders of Consciousness (DOC) are defined as disordered consciousness lasting longer than 28 days following severe brain injury, presenting a profound clinical and societal challenge. While advances in medical care have increased survival rates following severe brain injury, a significant number of patients fail to achieve meaningful recovery of consciousness. This persistent neurological impairment exerts profound multidimensional impacts, severely compromising patients’ quality of life and simultaneously imposing considerable socioeconomic burdens on caregivers and healthcare systems (1). The assessment of these patients predominantly relies on standardized behavioral scales, such as the Coma Recovery Scale-Revised (CRS-R). Conventional therapeutic strategies, including pharmacological and behavioral interventions, have demonstrated limited efficacy, particularly in the chronic stage of DOC. Therefore, the exploration of novel and effective therapeutic interventions is of paramount importance.

The neuropathophysiological underpinnings of DOC are increasingly understood as a dysfunction of large-scale brain networks, rather than isolated focal lesions. Critical among these is the disruption of the cortico-striatal-thalamic mesocircuit and the ascending reticular activating system, which are fundamental for arousal and awareness (2, 3). The thalamus, acting as a central hub for relaying sensory information and regulating cortical activity, is a lynchpin in the formation and maintenance of consciousness. Consequently, neuromodulation techniques aiming to restore consciousness often seek to upregulate excitatory thalamic output and re-establish network-level communication, promoting the recovery of behavioral responsiveness (4, 5). While interventions such as amantadine, transcranial direct current stimulation (tDCS) and repetitive transcranial magnetic stimulation (rTMS) have shown some promise, particularly in sub-acute DOC patients (4, 6), their utility in chronic patients is less established. In addition, these modalities face inherent limitations: amantadine lacks target specificity due to its systemic effects, whereas tDCS and rTMS, although non-invasive, are largely confined to modulating superficial cortical regions, lacking the capability to directly and precisely engage deep subcortical structures such as the thalamus.

To overcome these limitations, transcranial focused ultrasound (tFUS) has emerged as a groundbreaking non-invasive neuromodulation modality. Its unique ability to deliver focused acoustic energy with high spatial precision (on the order of millimeters) to deep brain structures makes it an ideal candidate for targeting the thalamus in chronic DOC patients. Initial pioneering studies have demonstrated the feasibility, safety, and potential neuro-excitatory effects of tFUS. For instance, a landmark case report showed behavioral improvements following a single session of thalamic stimulation (7). Subsequent pilot studies and case series have corroborated these findings, reporting temporary gains in behavioral responsiveness in some acute and chronic DOC patients (8–10). However, the current body of evidence is predominantly based on single-session or short-term interventions. The cumulative therapeutic effects, long-term safety, and durability of repeated, multi-week tFUS administration remain largely unexplored. Furthermore, the precise network-level mechanisms by which thalamic tFUS promotes recovery—including alterations in functional connectivity and neurovascular coupling—are not yet fully elucidated.

Herein, we present the case of a patient with chronic DOC who underwent a 4-week therapeutic regimen of neuronavigation-guided tFUS targeting the central thalamus. The primary objective of this study was to investigate the cumulative behavioral effects and safety of this prolonged intervention. Furthermore, by employing a multimodal assessment strategy combining resting-state functional near-infrared (fNIRS) and electroencephalogram (EEG), we aimed to longitudinally track changes in cerebral hemodynamics and neural oscillations. Through this comprehensive approach, we sought to provide novel insights into the network-level mechanisms underlying tFUS-mediated recovery of consciousness.

## Case presentation

### Patient history and previous treatment

A 46-year-old man presented to the emergency department with an acute onset of headache, dizziness, nausea, and vomiting, which rapidly progressed to a DOC. His past medical history was significant for hypertension and chronic use of tobacco and alcohol, with no known drug or food allergies. A nonenhanced computed tomography scan revealed a left thalamic hemorrhage with intraventricular extension and minor midbrain involvement. Emergency surgical intervention with burr-hole drainage was performed. His postoperative course was complicated by a prolonged coma and a secondary pulmonary infection, which necessitated a tracheotomy and anti-infective therapy. After his condition stabilized, he was discharged following 4 weeks of hospitalization. Over the subsequent 3 months, he received comprehensive rehabilitation, including physical and occupational therapy, acupuncture, and electrical stimulation. This resulted in only marginal improvement; he exhibited minimal voluntary movement in the left extremities and remained unable to follow commands. Upon admission to our department for the present study, he was diagnosed as being in a minimally conscious state with a CRS-R score of 11 (Auditory=2, Visual=3, Motor=5, Oromotor=1, Communication=0, Arousal=0).

### Ultrasound stimulation therapy regimen

**Figure 1:**
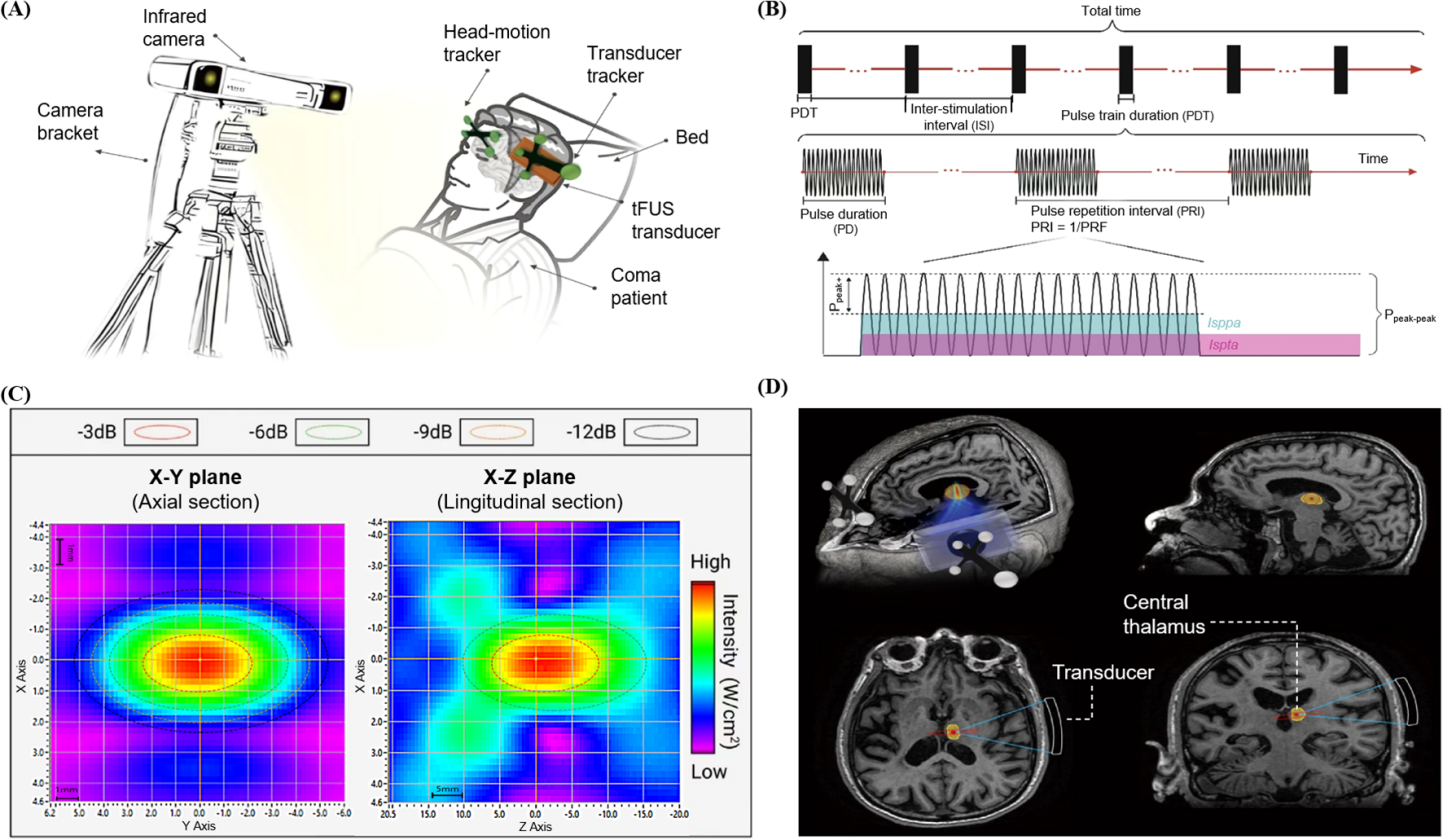
Experimental setup and targeting procedure for thalamic tFUS. **(A)** The tFUS system, comprising a control workstation for neuroimage visualization and treatment planning, and an optical navigation system (OpticalPos) with an infrared camera for real-time co-registration of the patient’s head and the transducer. **(B)** Schematic diagram of sonication pulse sequence and parameters. **(C)** The pressure profile is shown in the transverse (XY) planes at the acoustic focus and in the longitudinal (YZ) plane along the beam path. Colored circles denote changes in the acoustic field’s energy distribution. **(D)** MRI-based neuronavigation for targeting the right central thalamus, illustrating the planning of the acoustic trajectory from the scalp entry point to the intended target on the structural MRI of the patient.

In addition to a long-term, stable regimen of wakefulness-promoting treatments (amantadine, multisensory stimulation) established prior to the initiation of the tFUS protocol, the patient was administered 20 minutes of tFUS three times per week for four weeks (12 sessions in total). The treatment employed a single-element ultrasonic probe (GreenValley BrainTech Medical Technology Co. Ltd.) with a rectangular aperture of 80mm × 45mm and a focusing curvature of 85mm (**Figure 1A**). Free-field measurements using a hydrophone system (Precision Acoustics Ltd; UMS3 scanning system with NH0200 hydrophone, 0.2 mm element diameter) experimentally determined a -6 dB focal spot size of 3 mm (transverse-x) × 5 mm (transverse-y) × 25 mm (axial-z) (**Figure 1C**). The transducer was driven by an electronic control system to produce pulses with an operating frequency of 650kHz, a pulse duration (PD) of 300 μs, a pulse repetition interval (PRI) of 5 ms, yielding a pulse repetition frequency (PRF) of 200 Hz and a duty cycle (DC) of 6%. The pulse train duration (PTD) was set to 1 s, delivered every 6 seconds with a 5-second inter-stimulation interval (ISI) (**Figure 1B**). The incident peak-to-peak pressure amplitude at the focal point measured 1.2MPa, resulting in a spatial-peak pulse-average acoustic intensity (Isppa) of 12W/cm^2^, a spatial-peak time-average acoustic intensity (Ispta) of 720mW/cm^2^, a mechanical index (MI) of 0.74 and a cranial thermal index (TIC) of 1.6.

### Ultrasound stimulation navigation

The tFUS procedure was guided by an optical neuronavigation system (OpticalPos, Version 1.0.3, GreenValley BrainTech Medical Technology Co. Ltd, Shenzhen, China). Prior to the intervention, a high-resolution T1-weighted MPRAGE structural MRI was acquired for anatomical guidance (TR = 2530 ms, TE = 2.98 ms, TI = 1100 ms, Voxel size = 1.0 × 1.0 × 1.0 mm3). On the day of treatment, the patient was positioned comfortably in a supine position, with the head resting in a natural, unrestrained manner. The acquired MR images were imported into the built-in navigation software. The stimulation target, the right central thalamus, was then manually delineated based on the patient individual anatomy. This unilateral target was specifically selected to circumvent the primary lesion, which presented as a left thalamic hemorrhage. The decision to target the non-lesioned hemisphere was grounded in prior evidence suggesting that unilateral stimulation of an intact thalamic hub can effectively modulate bilaterally distributed networks (9). The software provided real-time visualization of the projected acoustic focus (**Figure 1D**, red sector) and the ultrasound beam path (**Figure 1D**, blue spindle) superimposed on the patient MRI. It is important to note that this visualization represents a free-field estimation and does not account for the phase aberration, attenuation, or refraction effects introduced by the individual’s skull. While the operator performed coordinate co-registration and subsequent transducer alignment, the patient was instructed to keep their head completely still. The operator manually adjusted the transducer position and orientation until the virtual focus was precisely aligned with the delineated target. Once the optimal position was achieved, the transducer was secured by a mechanical holder to maintain its position relative to the patient cranium throughout the session. To ensure optimal acoustic coupling, ultrasound gel was generously applied to eliminate any air gaps between the transducer and the scalp. Furthermore, care was taken to orient the incident acoustic beam as perpendicularly as possible to the local curvature of the skull to minimize beam refraction.

### Results for CRS-R and GCS scores

**Figure 2:**
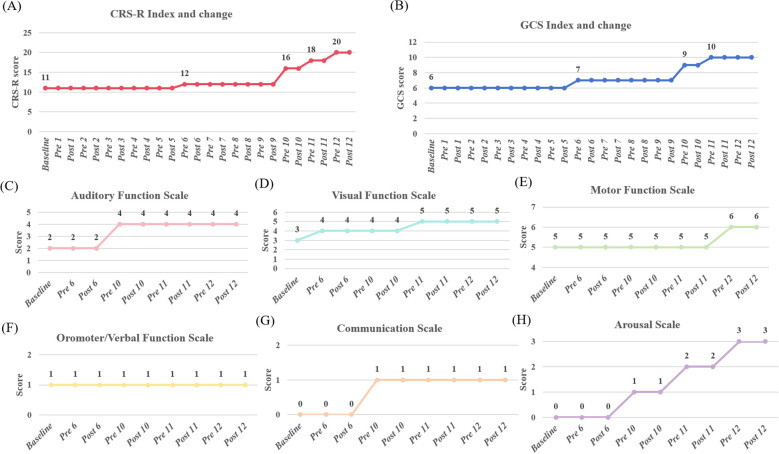
Changes in the CRS and GCS across tFUS sessions. **(A)** Evolution of the total CRS-R score. **(B)** Evolution of the GCS score. Baseline was assessed 1 day prior to the intervention. Pre 1 and Post 1 refer to assessments immediately before and after the first tFUS session, respectively, with subsequent time points following this pattern. **(C-H)** Evolution of scores for the individual CRS-R subscales: Auditory, Visual, Motor, Oromotor/Verbal Function Subscale, Communication and Arousal, throughout the tFUS treatment period.

Central thalamic tFUS administered with these parameters showed no observable alterations in vital parameters (blood pressure, heart rate, and blood oxygen). Cranial CT imaging revealed no gross structural abnormalities or signs of acute hemorrhage, and no adverse events were reported. These findings provide a rationale for the safety of focused ultrasound stimulation of the thalamus in the treatment of chronic DOC. Following the tFUS intervention, the patient displayed a clinical increase in behavioral responsiveness, emerging from a minimally conscious state. Specifically, the CRS-R total score increased from 11 at baseline to 20 (4 + 5 + 6+1 + 1+3) (**Figure 2A**), and the GCS score improved from E1VTM5 to E4VTM6 (**Figure 2B**). Analysis of the CRS-R subscales showed that except for the Oromotor/Verbal Function Subscale, the scores of all other subscales increased to varying degrees. In particular, the scores of the Auditory, Visual, Motor function and Arousal Subscale all reached the normal level after tFUS treatment (**Figures 2C-H**).

### Results for near-infrared brain function analysis

**Figure 3:**
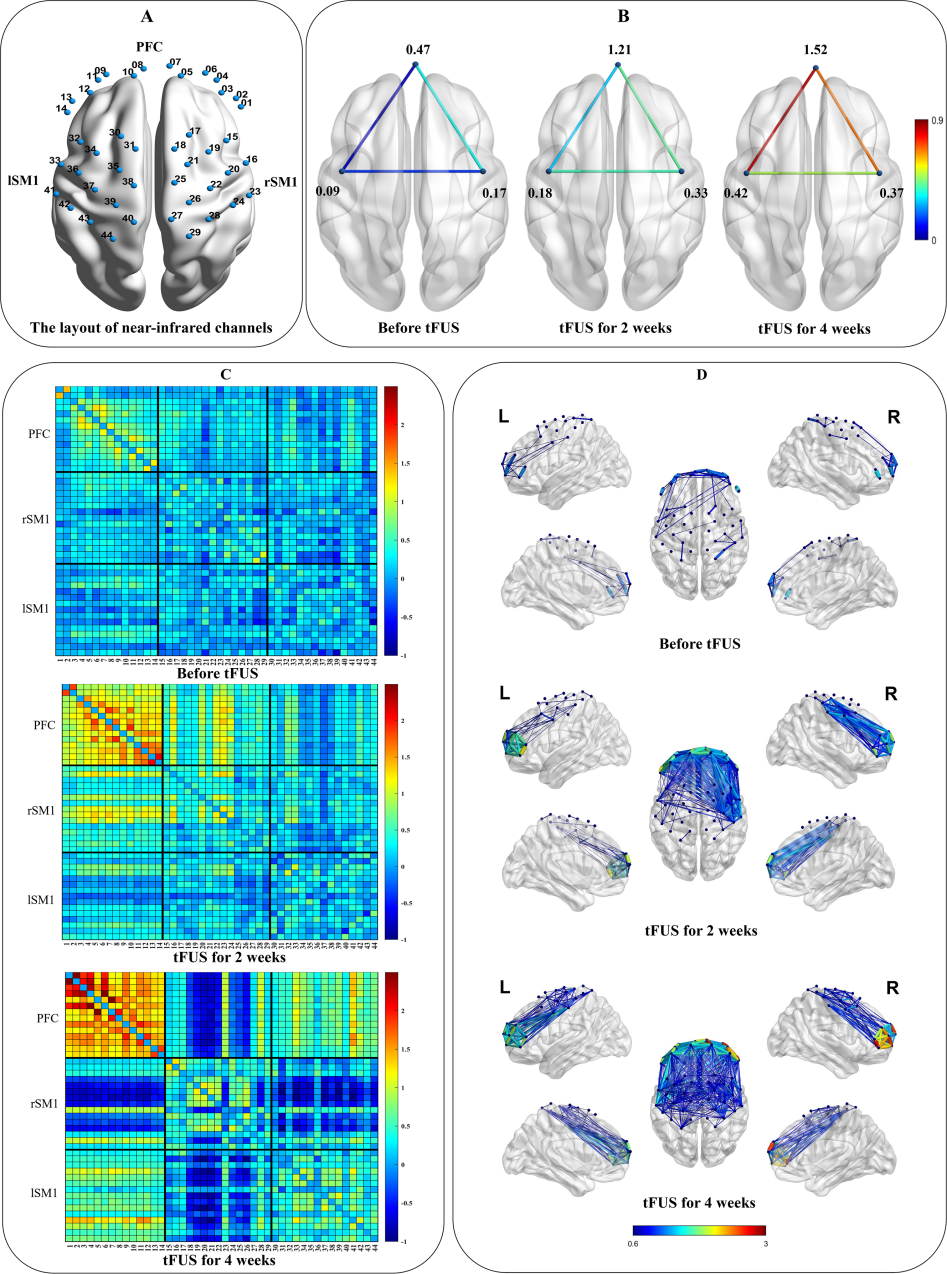
**(A)** The layout of the NIR channels in the bilateral prefrontal cortex (PFC: channels 1-14) and right and left primary sensorimotor cortex (rSM1: channels 15-29; lSM1: channels 30-44). **(B)** ROI-wise functional connections between the PFC and SM1 and within ROI. **(C)** Pair-wise functional connectivity in 2D. **(D)** Pair-wise functional connectivity in 3D.

To longitudinally assess cortical functional dynamics, we acquired ten-minute eyes-closed resting-state functional near-infrared spectroscopy (fNIRS) data at baseline, two weeks (6 sessions), and four weeks (12 sessions) of FUS intervention, utilizing a channel layout that covered key cortical association areas (**Figure 3A**). Data processing was conducted using the Homer2 and NIRS-KIT toolboxes within MATLAB 2022b (The MathWorks Inc., Massachusetts) (11). The analysis pipeline included motion correction via the Temporal Derivative Distribution Repair algorithm, followed by bandpass filtering of the hemodynamic signals between 0.01 and 0.08 Hz. Changes in oxyhemoglobin concentration were then used to compute functional connectivity matrices representing the pairwise correlations between all channels. For a higher-level network analysis, channels were grouped into three regions of interest (ROIs): the bilateral prefrontal cortex (PFC; channels 1-14), the right primary sensorimotor cortex (rSM1; channels 15-29) and the left primary sensorimotor cortex (lSM1; channels 30-44).

The resulting connectivity patterns, visualized using the Brain Net Viewer (11), revealed a progressive and substantial enhancement of cortical network integration over the course of the treatment. Specifically, quantitative analysis demonstrated a marked increase in both inter- and intra-regional functional connectivity. Cross-regional communication escalated between all key ROIs: connectivity between the PFC and rSM1 increased from 0.34 at baseline to 0.69 at 4-week tFUS, while the PFC-lSM1 pathway showed a robust surge from 0.03 to 0.86. Similarly, the coupling between the rSM1 and lSM1 strengthened from 0.14 to 0.48 (**Figures 3B, C**). Concurrently, internal network cohesion improved significantly across all three ROIs. The mean internal connectivity within the PFC exhibited the most pronounced enhancement, rising from 0.47 to 1.52, accompanied by increases within the lSM1 (0.09 to 0.42) and rSM1 (0.17 to 0.37) (**Figure 3B**). Notably, this neuroplastic effect followed a distinct spatiotemporal pattern. It initially emerged in the right hemisphere, ipsilateral to the ultrasound stimulation, with a more pronounced increase in connectivity strength and density observed at the 2-week mark. This effect subsequently expanded, leading to a more bilateral integration by the 4-week assessment (**Figure 3D**). This finding suggests that the tFUS intervention not only induced localized changes but also drove a broader, targeted reorganization of the cortical network.

### Results for EEG power spectral analysis

To longitudinally track neural dynamics, continuous EEG signals were recorded for 10 minutes at each assessment point using a 64-channel amplifier (ANT Neuro EEGO). Electrodes were arranged according to the International 10–20 Configuration, with CPz serving as the online reference, while and AFz as the ground. Vertical electrooculographic activity was monitored via an electrode positioned below the left eye to detect eye blinks. Data acquisition was performed at a sampling rate of 500 Hz, with all electrode impedances maintained below 5 kΩ. Offline preprocessing and analysis was performed in the Matlab environment using EEGLAB toolbox. The continuous data were first low-pass filtered at 70Hz and then high-pass filtered at 1Hz. A notch filter (48–52 Hz) was applied to remove power-line noise (50 Hz signal). Following this, the data was downsampled to 250Hz and epoched into non-overlapping 2-second segments. Noise-laden epochs were rejected through visual inspection, and noisy channels were replaced using spherical interpolation. An independent component analysis was then employed to identify and remove stereotypical artifacts, including eye blinks, muscle activity and cardiac signals. Finally, any remaining epochs containing amplitudes exceeding ±100 μV were discarded, and the data were re-referenced to the common average to produce clean segments for power spectral analysis. Negative values in the spectral plots arise from logarithmic scaling.

**Figure 4:**
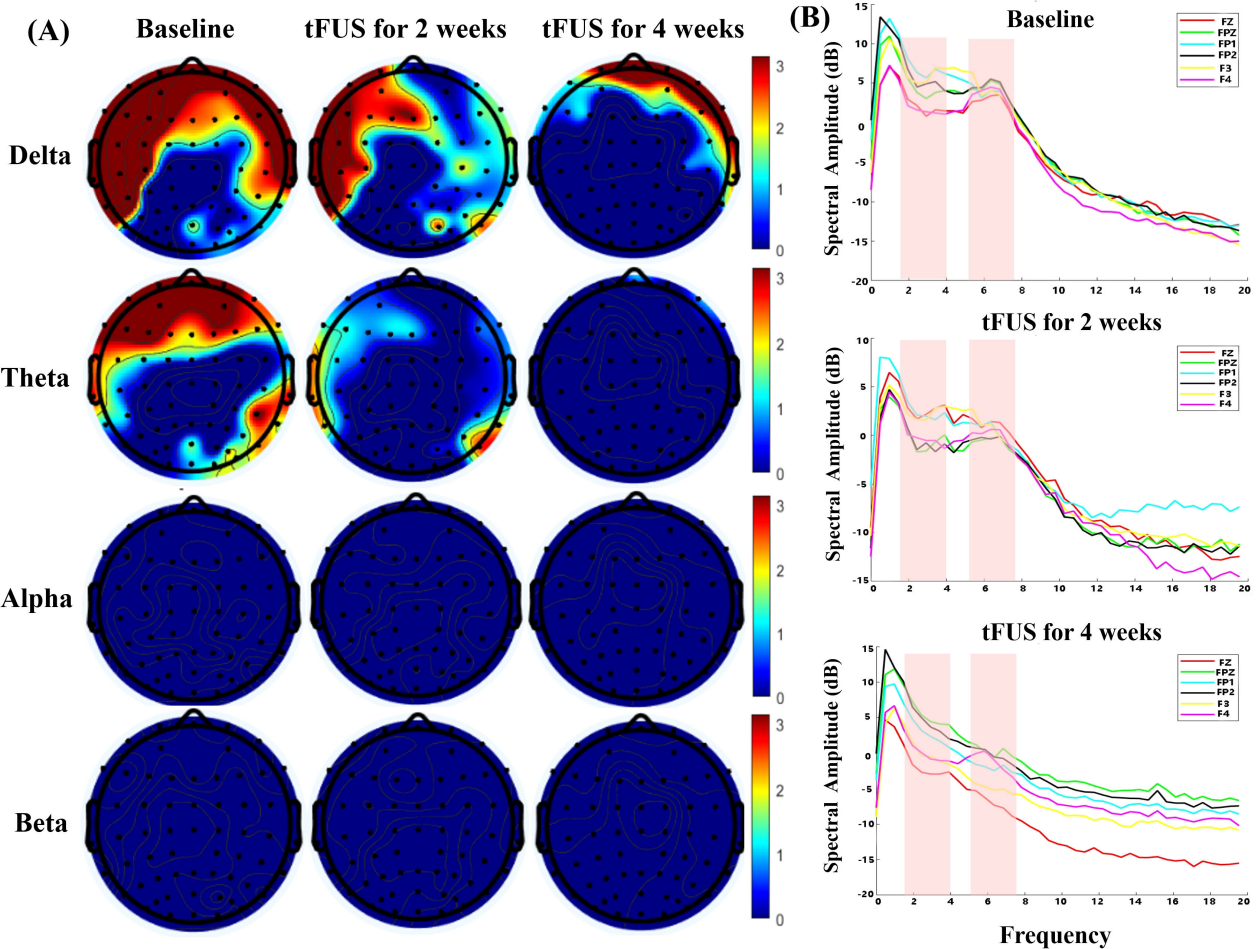
**(A)** Topography of power spectral density (dB) across different frequency bands. The numbers on the color bars represent the magnitude of Power Spectral Density (PSD) in decibels (dB). The color scale (0–3 dB) highlights high-power pathological activity. **(B)** Spectrograms of prefrontal electrodes. Pink shaded regions indicate the mainly Delta and Theta bands.

Longitudinal analysis of EEG revealed a progressive and marked modulation of brain activity over the four-week tFUS intervention period, particularly within the slow-wave frequency bands. Topographical power maps (**Figure 4A**) at baseline demonstrated prominent slow-wave activity, characterized by excessive power in the Delta (1-4Hz) and Theta (4-8Hz) bands predominantly over bilateral prefrontal regions, a typical neurophysiological signature of impaired consciousness. The color scale for these topographical maps was set to emphasize high-power pathological oscillations, displaying positive dB values while suppressing low-power background activity. Following two weeks of tFUS, the spatial extent and magnitude of this pathological slow-wave activity began to recede, a trend that became substantially more pronounced by the four-week mark, where frontal Delta and Theta power had diminished significantly. These topographical changes were quantitatively corroborated by power spectral density analysis of key prefrontal electrodes (**Figure 4B**). The spectral plots in the pink shaded regions clearly show a systematic, session-by-session decrease in the amplitude of both Delta and Theta peaks across the treatment timeline. Specifically, Delta power attenuated from 4.87 dB at baseline to 2.14 dB at week 4, while Theta power dropped from 3.35 dB to -0.01 dB. Regarding the higher frequency bands, while a mild concomitant increase was observed in Alpha (8–13 Hz) and Beta (13–30 Hz) power—potentially reflecting a renormalization of cortical arousal—these changes were less pronounced than the suppression of pathological slow-wave oscillations. These findings collectively suggest that the tFUS intervention selectively suppressed aberrant frontal slow-wave oscillations associated with the patient’s chronic disorder of consciousness.

### Multimodal results integration analysis

The clinically significant behavioral recovery observed in the patient was strongly mirrored by corresponding neurophysiological and hemodynamic changes, providing a cohesive, multi-modal account of tFUS-induced neuroplasticity. The progressive suppression of pathological slow-wave oscillations in the prefrontal cortex, as captured by EEG, occurred concomitantly with a robust enhancement of functional connectivity within this same region, revealed by fNIRS. This electrophysiological normalization, characterized by the dampening of aberrant Delta and Theta activity, created a neural environment conducive to the restoration of effective large-scale network communication. The fNIRS data further elucidated this process, demonstrating not only a strengthening of local prefrontal circuits but also a re-establishment of long-range connections to sensorimotor areas. Together, these profound neural reorganizations provide a plausible neurobiological substrate for the patient’s remarkable clinical trajectory, where the objectively measured improvements in arousal, motor function, visual and auditory processing directly reflect the underlying restoration of cortical integration and communication. This convergence of evidence across behavioral, metabolic, and electrophysiological domains suggests that thalamic tFUS may modulate cortical network activity. These observed physiological changes parallel a cascade of restorative events associated with the patient’s meaningful emergence from a minimally conscious state.

## Discussion

This case explored the effectiveness and safety of 12 sessions of tFUS applied to the right central thalamus in an individual with chronic DOC under the guidance of neuronavigation. The transcranial focused ultrasound parameters in this study were well below the FDA recommendation of a maximum ISPPA of 190 W/cm^2^ and a maximum ISPTA of 0.72 W/cm^2^. Furthermore, the protocol adhered to the biophysical safety guidelines established in the recent ITRUSST consensus statement (12), maintaining a MI (0.74) significantly below the recommended threshold of 1.9 and ensuring a theoretical temperature rise of less than 2 °C.

The clinically meaningful behavioral recovery observed in this patient provides preliminary evidence supporting the therapeutic potential of multi-session thalamic tFUS in chronic DOC. This outcome aligns with and extends the pioneering work initiated by Monti and subsequently advanced by Cain and colleagues (7–10), who first established thalamic neuromodulation as a viable strategy. Our patient’s emergence from a minimally conscious state, marked by a substantial increase in the CRS-R score from 11 to 20 and the consistent ability to follow commands and use objects, suggests a potential treatment effect. A noteworthy observation from our longitudinal intervention is the apparent cumulative nature of this benefit. While Monti’s seminal 2016 case described immediate behavioral gains, subsequent investigations by the same group involving repeated stimulation sessions have also suggested a potential cumulative effect (8–10). Our study aligns with and extends this emerging evidence, demonstrating that in the chronic setting, progress materialized gradually over the course of twelve treatment sessions, whereas no significant behavioral changes were observed within the first hour following any individual sonication session. Notably, neurophysiological improvements (fNIRS/EEG) emerged after just two weeks (six sessions), preceding the substantial behavioral recovery observed in the final two weeks. This temporal dissociation suggests that neural changes may need to accumulate to a critical threshold before manifesting as overt clinical improvements. This observation suggests a potential action in the chronic setting. While tFUS may facilitate immediate network re-engagement in the post-acute brain, we hypothesize that its therapeutic impact in chronic states might rely on cumulative neurophysiological changes. This hypothesis is consistent with preclinical evidence showing that repeated ultrasound stimulation can upregulate brain-derived neurotrophic factor (BDNF) and modulate synaptic strength (13, 14). Besides, we must critically evaluate the potential contribution of concomitant treatments, specifically the ongoing administration of amantadine and multisensory stimulation. While these interventions were maintained at stable dosages and frequencies prior to and during the study, preventing them from explaining the sudden inflection in recovery, their potential synergistic interaction with tFUS remains an important consideration. Moreover, a six-month video follow-up confirmed sustained clinical gains, with the patient remaining conscious and responsive to commands, suggesting that the observed changes may represent a lasting shift rather than transient modulation.

The fNIRS data provide a potential neurobiological correlate for the observed clinical improvement of the patient, indicating that thalamic tFUS was associated with enhanced cortical functional connectivity. Our observation that connectivity enhancement was most pronounced in the prefrontal cortex aligns with the established understanding of DOC as a disconnection syndrome, where frontal network integrity is critically impaired (15, 16). This finding contributes to a growing body of evidence suggesting that diverse neuromodulatory techniques (11, 17, 18), including vagus nerve stimulation and deep brain stimulation, converge on a common mechanistic pathway: the reactivation of coherent brain-wide communication. While these studies collectively validate the principle of enhancing connectivity for DOC treatment, our results offer a more granular insight that distinguishes the action of tFUS. The distinct spatiotemporal evolution of network recovery, beginning in the hemisphere ipsilateral to the thalamic stimulation and subsequently expanding to achieve bilateral integration, provides a fascinating window into the neuromodulatory cascade. This suggests that tFUS does not merely induce a diffuse, global increase in brain activity. Instead, it appears to initiate a targeted, propagating wave of neuroplasticity. This finding underscores the unique capacity of focused ultrasound to selectively engage a critical subcortical node and trigger a structured, downstream reorganization of the cortical functional architecture, a mechanism that may be fundamental to its therapeutic effect in chronic brain injury.

The longitudinal EEG analysis offers electrophysiological insights for the recovery of the patient, showing a targeted reduction of the aberrant slow-wave activity that is a well-established hallmark of severe brain injury. Our primary finding that clinical improvement is associated with the normalization of prefrontal Delta and Theta power positions thalamic tFUS alongside other effective neuromodulatory interventions, such as intermittent theta burst stimulation, that have been linked to suppression of pathological oscillations (16). Although excessive slow-wave activity is consistently reported in DOC (19–22), our data document its progressive reduction in association with targeted subcortical stimulation. Intriguingly, the therapeutic effect was characterized by a distinct reciprocal pattern: a suppression of pathological slow-wave bands accompanied by a concurrent and mild re-emergence of Alpha and Beta frequencies. This spectral shift is a critical finding. It suggests that thalamic tFUS acts primarily by disrupting the dominant, low-frequency network dynamics that inhibit cortical function. By effectively quieting this pervasive slow-wave “noise,” the intervention appears to create a permissive electrophysiological environment that facilitates the renormalization of physiological high-frequency rhythms, which are essential for global cortical arousal and information processing.

This case study provides a multi-modal description of recovery, where the convergence of behavioral, hemodynamic, and electrophysiological data offers a mechanistic window into how targeted thalamic neuromodulation can reverse pathological signatures of chronic brain injury. The central thalamus, as a critical hub within the ascending reticular activating system, plays an indispensable role in maintaining cortical arousal by coordinating activity across large-scale brain networks (23). Our findings suggest that tFUS effectively leveraged this strategic position. The primary proposed mechanism involves mechanical effects, whereby low-intensity acoustic pressure waves may act upon mechanosensitive ion channels within the neuronal membrane, directly altering ionic flux and, consequently, neuronal excitability and synaptic transmission (24, 25). This direct, non-thermal modulation of thalamic neuronal firing provides a plausible biophysical trigger for the observed downstream cascade. Specifically, by altering the firing patterns within the thalamus, tFUS appears to have rebalanced pathological thalamocortical dynamics, leading to the profound suppression of aberrant frontal Delta and Theta slow-wave activity observed in our EEG data. By lifting this inhibitory brake, tFUS created a permissive neurophysiological environment for the re-emergence of coherent high-frequency rhythms and metabolically demanding network activity, as evidenced by the robust enhancement of functional connectivity in our fNIRS results (26). Moreover, the restorative process is likely multifactorial, as preclinical studies have suggested that tFUS can also confer neuroprotective benefits by enhancing cerebral blood flow, promoting the clearance of metabolic waste via the glymphatic system, and reducing neuroinflammation (27, 28). Therefore, the integrated findings paint a cohesive picture: precise tFUS of the thalamus initiates a therapeutic cascade via direct mechanotransduction, which in turn normalizes cortical rhythms, allows dormant large-scale networks to resume communication through a combination of neuroplastic and physiological enhancements, and ultimately enables the return of observable, conscious behavior. These observations, while limited to a single case, provide preliminary evidence warranting further controlled investigation.

## Limitations

The inferential power of this study is inherently constrained by its single-case design. While the multi-modal evidence presented is persuasive and internally consistent, the remarkable recovery observed in this individual cannot be generalized to the heterogeneous population of patients with chronic DOC. Regarding the natural history of the disease, we acknowledge that while late recovery is possible, epidemiological data indicate that meaningful functional improvement is significantly less frequent in chronic hemorrhagic etiologies compared to traumatic cases (29). Specifically, longitudinal studies have reported that only a small minority of patients with chronic anoxic brain injury recover consciousness after three months, with probabilities estimated to be as low as < 17% in similar chronic timeframes (30). The patient had exhibited a stable clinical plateau with no signs of awakening for 4 months prior to enrollment, yet suggested a clear trajectory of improvement that was temporally time-locked to the four-week tFUS intervention window. The temporal coincidence with the intervention makes spontaneous recovery less probable given the prior prolonged stability. To rigorously establish this relationship and control for potential placebo effects, a randomized, double-blind, sham-controlled clinical trial is imperative. We acknowledge this necessity, and such a trial is presently being undertaken by our group to systematically validate these promising findings. A further methodological limitation relates to the assumptions regarding transcranial acoustic propagation and the absence of patient-specific acoustic simulations. In this study, targeting relied on neuronavigation assuming homogeneous skull acoustic properties and ideal wave propagation, without individual correction for skull-induced phase aberrations and attenuation. As demonstrated by Cain et al., such aberrations can distort or displace the ultrasound focus toward adjacent structures (e.g., globus pallidus), potentially affecting target engagement and dosimetry accuracy despite precise anatomical navigation (31). Future multi-patient trials must incorporate numerical acoustic modeling (e.g., k-Wave based on individual CT data) to rigorously correct for these skull effects, ensuring precise targeting accuracy and dosimetry. An additional limitation concerns the interpretation of the EEG findings. The analysis focused primarily on prefrontal electrodes, as global topography revealed the most pronounced and consistent longitudinal reductions in slow-wave power in prefrontal regions, whereas posterior parietal regions showed no distinct temporal changes in this patient. This regional prioritization was chosen to highlight the most robust physiological signals linked to clinical recovery in this single-case report. Nevertheless, a comprehensive understanding of tFUS effects on consciousness-related circuits requires simultaneous assessment of both prefrontal and posterior parietal networks in our ongoing randomized controlled trial.

## Conclusion

In conclusion, this case report provides preliminary multi-modal evidence suggesting that multi-session, low-intensity tFUS targeting the central thalamus may facilitate the recovery of consciousness in a patient with a chronic DOC. We observed a tight coupling between the clinical emergence, a progressive reintegration of large-scale cortical networks measured by fNIRS, and a concurrent normalization of pathological slow-wave oscillations captured by EEG. These convergent findings suggest a plausible therapeutic cascade wherein targeted, non-invasive neuromodulation of a critical subcortical hub may help modulate core pathophysiological features of the chronically injured brain. While acknowledging the clear limitations inherent to a single-case study, this work provides a strong mechanistic rationale for the continued, rigorous investigation of tFUS through larger, sham-controlled clinical trials to definitively establish its role as a novel intervention for restoring consciousness.

## Data Availability

The original contributions presented in the study are included in the article/supplementary material. Further inquiries can be directed to the corresponding author/s.
